# Extending the BEAGLE library to a multi-FPGA platform

**DOI:** 10.1186/1471-2105-14-25

**Published:** 2013-01-19

**Authors:** Zheming Jin, Jason D Bakos

**Affiliations:** 1 Department of Computer Science and Engineering, University of South Carolina, Columbia, SC, USA

## Abstract

**Background:**

Maximum Likelihood (ML)-based phylogenetic inference using Felsenstein’s pruning algorithm is a standard method for estimating the evolutionary relationships amongst a set of species based on DNA sequence data, and is used in popular applications such as RAxML, PHYLIP, GARLI, BEAST, and MrBayes. The Phylogenetic Likelihood Function (PLF) and its associated scaling and normalization steps comprise the computational kernel for these tools. These computations are data intensive but contain fine grain parallelism that can be exploited by coprocessor architectures such as FPGAs and GPUs. A general purpose API called BEAGLE has recently been developed that includes optimized implementations of Felsenstein’s pruning algorithm for various data parallel architectures. In this paper, we extend the BEAGLE API to a multiple Field Programmable Gate Array (FPGA)-based platform called the Convey HC-1.

**Results:**

The core calculation of our implementation, which includes both the phylogenetic likelihood function (PLF) and the tree likelihood calculation, has an arithmetic intensity of 130 floating-point operations per 64 bytes of I/O, or 2.03 ops/byte. Its performance can thus be calculated as a function of the host platform’s peak memory bandwidth and the implementation’s memory efficiency, as *2.03 × peak bandwidth × memory efficiency*. Our FPGA-based platform has a peak bandwidth of 76.8 GB/s and our implementation achieves a memory efficiency of approximately 50%, which gives an average throughput of 78 Gflops. This represents a ~40X speedup when compared with BEAGLE’s CPU implementation on a dual Xeon 5520 and 3X speedup versus BEAGLE’s GPU implementation on a Tesla T10 GPU for very large data sizes. The power consumption is 92 W, yielding a power efficiency of 1.7 Gflops per Watt.

**Conclusions:**

The use of data parallel architectures to achieve high performance for likelihood-based phylogenetic inference requires high memory bandwidth and a design methodology that emphasizes high memory efficiency. To achieve this objective, we integrated 32 pipelined processing elements (PEs) across four FPGAs. For the design of each PE, we developed a specialized synthesis tool to generate a floating-point pipeline with resource and throughput constraints to match the target platform. We have found that using low-latency floating-point operators can significantly reduce FPGA area and still meet timing requirement on the target platform. We found that this design methodology can achieve performance that exceeds that of a GPU-based coprocessor.

## Background

Different Bayesian and likelihood-based phylogenetic inference tools use various methods for generating a sequence of candidate trees, but in general these tools use the Phylogenetic Likelihood Function (PLF) to evaluate the likelihood of a proposed tree [1]. Equation 1 shows the PLF.

(1) $$\begin{array}{l} \begin{array}{c} L_{k}^{\left(i\right)}\left(s\right)\\ s\in \left(A,C,G,T\right) \end{array}=\left(\sum_{x\in \left(A,C,G,T\right)}\mathrm{Prob}\left(x|s,t_{l}\right)L_{l}^{\left(i\right)}\left(x\right)\right)\\ \;\times \left(\sum_{y\in \left(A,C,G,T\right)}\mathrm{Prob}\left(y|s,t_{r}\right)L_{r}^{\left(i\right)}\left(y\right)\right) \end{array}$$

The computational components described in this paper target the nucleotide model of evolution, but the design methodology can be generalized to all discrete-character models. The PLF computes the conditional likelihood of each of the four bases being at position *i* in an ancestral sequence as a function of the conditional likelihoods of each of the bases at the same position in the left and right descendent nodes. Once all conditional likelihoods are computed for a candidate tree, the tree likelihood can be computed as a function of the conditional likelihoods at the root node, as shown in Equation 2. Though scaling in the equation is not part of the mathematical algorithm of the PLF, it is part of a computational algorithm which implements the PLF as a means to cope with limited numerical precision and large trees.

(2) $$\begin{array}{l} \sum_{i}\left(\left(\log\left({}_{j}^{\max}L_{0}^{\left(i\right)}\left(j\right)\right)+\mathrm{scale}r_{i}+\log\right)\right)\\ \;\times \left(\left(\left(\sum_{s\in \left(A,C,G,T\right)}\pi_{s}\frac{L_{0}^{\left(i\right)}\left(s\right)}{{}_{j}^{\max}L_{0}^{\left(i\right)}\left(j\right)}\right)\right)\times \mathrm{nSite}s_{i}\right) \end{array}$$

Computing the PLF and tree likelihood for candidate trees comprises the computational kernel of these tools. Since multiple tools use a common likelihood computation, Ayres et al., implemented a finely tuned implementation of the likelihood computation as a general-purpose library called BEAGLE [2]. BEAGLE supports CPU and GPU-based architectures, but does not yet support Field Programmable Gate Array (FPGA)-based architectures such as the Convey HC-1 [3]. In this paper, we describe our effort to add FPGA support to BEAGLE as well as the resultant performance.

### Related work

There has been previous work in accelerating the PLF to FPGA-based coprocessor architectures. Mak and Lam were perhaps the first team to implement likelihood-based phylogeny inference on an FPGA [4]. They used special-purpose logic in the FPGA fabric to perform the PLF using fixed-point arithmetic. Alachiotis et al. also implemented the PLF in special purpose logic and achieved an average speedup of four relative to software on a sixteen core processor [5,6].

There has also been recent work in using Graphical Processor Units (GPUs) as co-processors for ML-based phylogenetic inference. In recent work, Suchard et al. used the NVIDIA CUDA framework [7] to implement single and double precision versions of the PLF [8]. Using three NVIDIA GTX280 GPUs, they achieved a speedup of 20 for the single precision nucleotide model as compared to single-threaded software. Zhou et al. developed a GPU implementation and evaluated it on a CPU running four processes and two NVIDIA GTX 480 GPUs [9]. They achieved a speedup of 42.3 relative to MrBayes running on a CPU with a single thread, and 13.9 when compared to an optimized CPU version with 4 threads.

### Descriptions of PLF kernel

The kernel function of BEAGLE depends on which options and evolutionary models are used for the analysis. When using the 4-state nucleotide model, nearly all the execution time is spent evaluating the log-likelihood score. This evaluation consists of evaluating the PLF, normalizing the conditional likelihood tables to avoid numerical underflow, and updating two log-scaler arrays.

The kernel algorithm is described in C-style pseudocode below. Note that although our implementation produces consistent results with that of BEAGLE, the pseudocode describes the authors’ implementation and is not necessarily descriptive of the corresponding kernel included in BEAGLE.

### Pseudocode 1: BEAGLE Kernel

// for each nucleotide in the sequence perform the PLF to complete the four-column

// conditional likelihood table

h = 0;

for (k = 0; k < nsites; k++) {

State is {AA, AC, AG, AT, CA, CC, CG, CT, GA, GC, GG, GT, TA, TC, TG, TT};

Base is {A, C, G, T};

State = State.first;

for (i = 0; i < 4; i++) {

Base = Base.first;

sopL = sopR = 0;

for (j = 0; j < 4; j++) {

sopL = sopL + tipL[State] * clL[Base];

sopR = sopR + tipR[State] * clR[Base];

State = State.next;

Base = Base.next;

}

clP[h + i] = sopL * sopR;

}

// find the maximum of the previously computed values (scaler value)

scaler = 0;

for (i = 0; i < 4; i++)

if (clP[h + i] > scaler) scaler = clP[h + i];

// normalize the previously computed values

for (i = 0; i < 4; i++)

clP[h + i] = clP[h + i] / scaler;

// store the log of the scaler value and store in scP array

scP[k] = log(scaler);

// update the lnScaler array

lnScaler[k] = scP[k] * lnScaler[k];

// accumulate the log-likelihood value

condLike = 0;

for (i = 0; i < 4; i++)

condLike = condLike + bs[i] * clP[h + i];

lnL = lnL + numSites[k] * (lnScale[k] + log(condLike));

// increment counters

h = h + 4; clL = clL + 4; clR = clR + 4;

}

double log (double x) {

// initialize binary search

log_comp = −16;

coeff_set = 0;

coeff_incr = 8;

log_comp_incr = 8;

// perform a logarithmic binary search

for (i = 0;i < 4;i++) {

if (x < 10^log_comp) {

log_comp = log_comp - log_comp_incr;

} else {

log_comp = log_comp + log_comp_incr;

coeff_set = coeff_set + coeff_incr;

}

coeff_incr = coeff_incr / 2;

log_comp_incr = log_comp_incr / 2;

}

// compute the polynomial approximation

return_val = 0;

pow_x = 1.0;

for (i = 0; i < 5; i++) {

return_val + = return_val + coeff[coeff_set][i] * pow_x;

pow_x = pow_x * x;

}

return return_val;

}

The natural log approximation is implemented as an order-5 polynomial where the coefficients are divided into 16 segments whose range scales logarithmically. The coefficients are computed using a Chebyshev approximation [10]. In all of our experiments, we verify that the results computed from our design, including the log approximation, are accurate to within 1% of the results delivered by BEAGLE.

## Implementation

Our objective is to implement the PLF and tree likelihood computations on a Convey HC-1 heterogeneous platform as a hardware/software co-design. This application is a good match for the HC-1, which provides high memory bandwidth and its FPGAs are well-suited for computing data intensive loops containing no loop-carried dependences except for the accumulate required for the final likelihood value. We first briefly describe the platform then discuss our implementation in detail.

### Platform

**Figure 1:**
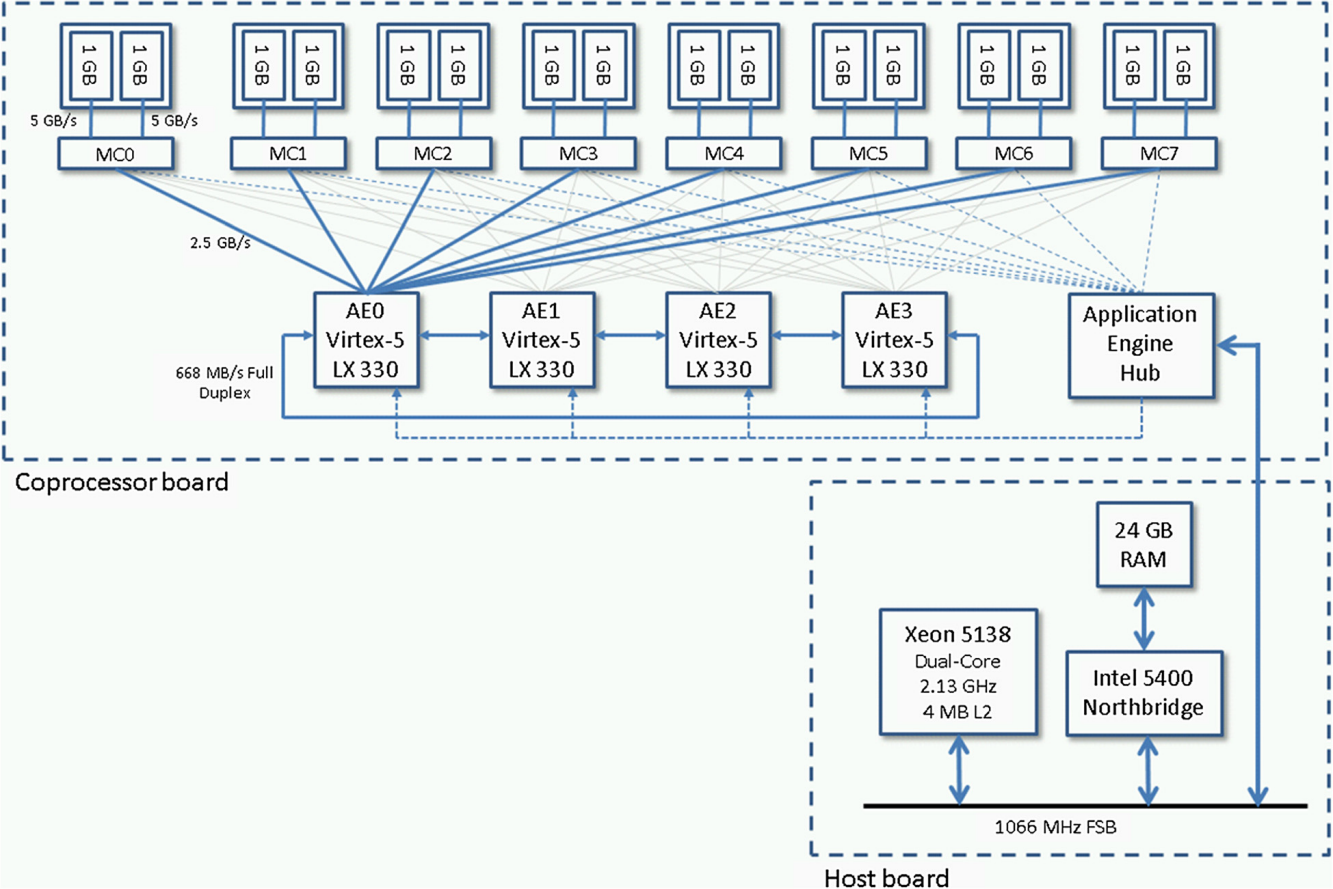
**The HC-1 coprocessor board.** Four application engines connect to eight memory controllers through a full crossbar.

Figure 1 shows the design of our target platform, the Convey HC-1. The Convey HC-1 is a reconfigurable computer containing an FPGA-based coprocessor attached to a host motherboard through a socket-based front-side bus interface. Unlike socket-based coprocessors from Nallatech [11], DRC [12], and XtremeData [13], which are confined to a footprint matching the size of the socket, Convey uses a mezzanine connector to bring the front side bus (FSB) interface to a large coprocessor board (roughly the size of an ATX motherboard).

The coprocessor board contains four user-programmable Virtex-5 LX 330 FPGAs called “application engines (AEs)”. The coprocessor board also contains eight memory controllers, each of which is implemented on its own Virtex-5 FPGA. Each of the AEs is connected to each of the eight memory controllers through a 4x4 crossbar switch on each memory controller.

Each memory controller allows up to two independent 64-bit memory transactions (read or write) per cycle on a 150 MHz clock, giving a peak theoretical bandwidth of 16 bytes * 150 MHz = 2.4 GB/s per memory controller, or 8 * 2.4 GB/s = 19.2 GB/s per AE, or 4 * 19.2 GB/s = 76.8 GB/s of aggregate bandwidth for the coprocessor board.

Although the HC-1’s coprocessor shares the same memory space as the host CPU, the coprocessor’s memory is partitioned into eight disjoint regions corresponding to each of the eight memory controllers. In other words, each memory region is only accessible by one of the eight memory interfaces.

Each of the eight memory controllers are connected to two Convey-designed scatter–gather DIMM (SG-DIMM) modules. Each of these two DIMMs contains eight DRAMs, each with eight decoupled DRAM banks that can be addressed independently. Each of the memory controllers attempts to maximize bandwidth by scheduling incoming memory requests to each of the sixteen banks, routing requests to non-busy banks and grouping reads and writes into bursts to minimize bus turns (state change between read and write). A reorder module, developed by Convey, re-arranges the loaded data to match the request order before delivering it back to the user logic.

The effectiveness of their scheduler depends on the memory access pattern issued by the user logic on the AEs. Contention for crossbar ports and bank request FIFOs cause the HC-1’s memory controller to throttle the AE by asserting a “stall” feedback signal. These stalls reduce the effective memory bandwidth.

For memory-bound kernels the user logic should attempt to access memory during every clock cycle of operation. Any cycle where the logic does not access memory is either due to inefficiency in the AE’s memory interface or due to a stall request from Convey’s memory controller. In order to calculate actual memory bandwidth, we define memory efficiency as:

$$\begin{array}{l} \mathrm{efficiency}=\left(\mathrm{number}\;\mathrm{of}\;\mathrm{memory}\;\mathrm{reads}\right)\\ \left(\;+\mathrm{number}\;\mathrm{of}\;\mathrm{memory}\;\mathrm{writes}\right)\\ \;/\left(\mathrm{number}\;\mathrm{of}\;\mathrm{execution}\;\mathrm{cycles}\right) \end{array}$$

Actual memory bandwidth can therefore be computed as:

$$\mathrm{actual}\;\mathrm{bandwidth}=\left(\mathrm{peak}\;\mathrm{bandwidth}\right)\times \mathrm{efficiency}$$

### Hardware implementation of the kernel

On the coprocessor, each AE has eight processing units (PEs). To achieve maximum memory efficiency, each should make full use of the 128-bit per cycle memory channel. Each PE implements the kernel described in Pseudocode 1 and thus each consumed 10 inputs per loop iteration (for each character in the input sequence), performing 130 single precision floating-point operations, and producing six outputs. Since the memory interface can only deliver four floating values per cycle, each PE requires three cycles to read its inputs and one and a half cycles to write its results for each input character.

### Resource and throughput constrained synthesis

The data introduction interval (DII) is the number of cycles required for the pipeline to read all of its inputs from the available input ports, i.e. $\mathrm{DII}=\left\lceil \frac{l}{p}\right\rceil$, where *l* = number of logical input ports and *p* = the number of physical input ports. In this case, the number of logical input ports is ten and the number of physical input ports is four. Given these parameters, our pipeline synthesis tool synthesizes this expression into an arithmetic pipeline having minimum DII, minimum critical path latency, and using the minimum number of floating-point functional units.

In conventional scheduling it is sufficient to provide at least one functional unit of each required functional unit type to ensure that a schedule exists. However, in order to achieve maximum throughput, the minimum number of functional units of an operation type is $R\geq \left\lceil \frac{M}{\mathrm{DII}}\right\rceil$, where *M* is the number of operators of a type in the DFG, and *DII* the data introduction interval.

We use the “As Soon As Possible (ASAP)” scheduling technique for data path synthesis. ASAP repeatedly schedules the ready operations to the time slot in a manner of first-come-first-served. The start time of each operation is the minimum allowed by the dependencies of operations.

Our pipeline synthesis tool takes, as input, the number of input ports of the target platform (physical input ports), the number of input variables derived from the target expression (logical input ports), and a data flow graph (DFG) representing the target expression. Given these parameters, the tool converts the DFG into a pipeline described in Verilog hardware description language with minimum number of floating-point functional units.

As an example, consider the high level code below, which computes one column of the conditional probability table.

clP[0] = (tipL[AA]*clL[A] + tipL[AC]*clL[C] + tipL[AG]*clL[G] + tipL[AT]*clL[T]) * (tipR[AA]*clR[A] + tipR[AC]*clR[C] + tipR[AG]*clR[G] + tipR[AT]*clR[T]);

The two 4x4 transition likelihood tables **tipL** and **tipR** are invariant across all nodes and are thus treated as constants. As such, this expression has eight inputs and one output.

**Figure 2:**
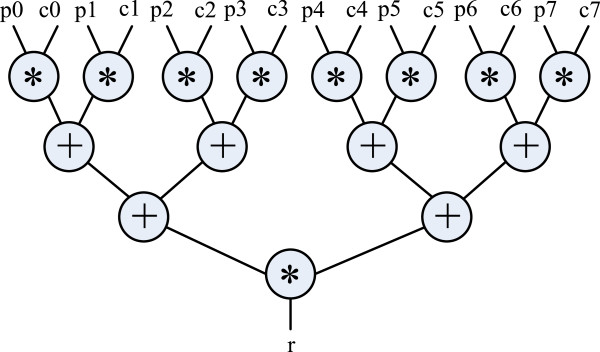
An arithmetic operation as a DFG.

**Figure 3:**
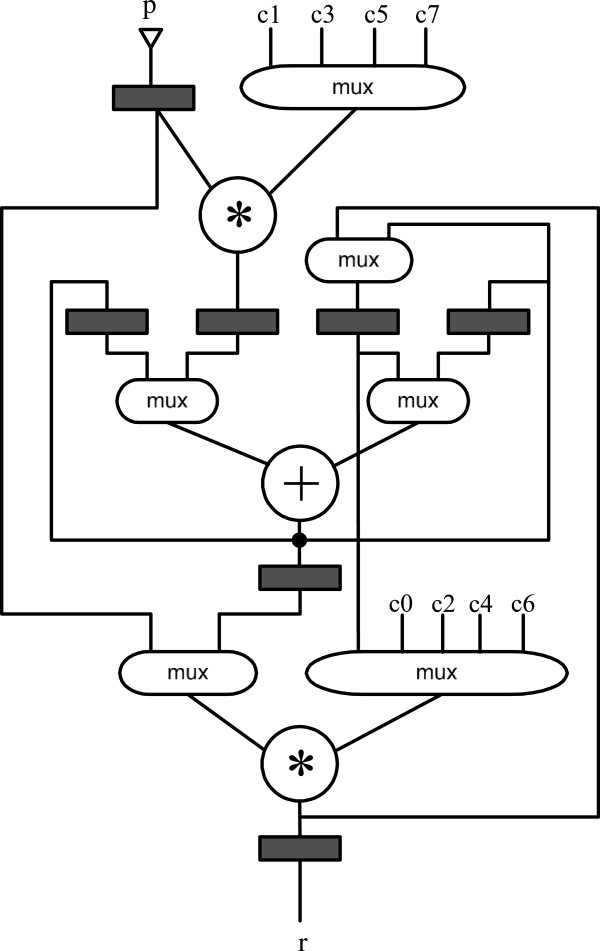
**Pipeline circuit generated from DFG in Figure **2**.**

The DFG for this expression is shown in Figure 2. If this expression is synthesized onto a platform that can only provide one input value per clock, it can be synthesized as shown in Figure 3 with only one adder and two multipliers. The multiplexers select different input data to feed the functional units. The dark-colored registers save the intermediate results of the functional units.

**Figure 4:**
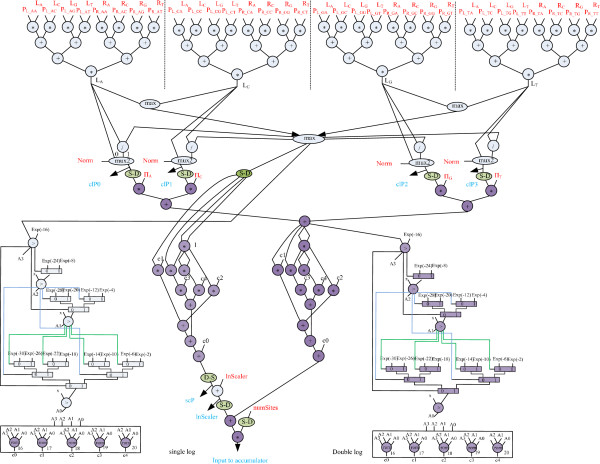
Full data flow graph of PLF and tree likelihood calculation.

The DFG of the full PLF kernel is shown in Figure 4. The number of floating-point operations in DFG is 38 additions, 55 multiplications, 4 divisions and 11 comparisons. Our tool’s resource sharing feature maps these arithmetic operations into 13, 19, 2 and 4 functional operators respectively. However, we find that it is infeasible to fit all the single-precision floating-point operators on one PE without adjusting the latency of each operator to reduce resource usage for the target device. By carefully evaluating the latency and resource usage of each operator in Xilinx CORE Generator System’s resource estimation [14], we used low-latency functional units for adder, multiplier and comparators. Using trial and error, we determined the latency of the floating-point divider needs to be around 11 to satisfy the 150 MHz timing constraint. Using low-latency operators we were able to reduce significantly FPGA’s slice registers for each functional unit and the entire pipeline, meeting the demanding resource requirement for each PE. Table 1 lists the low- and max-latency and the number of slice registers of each floating-point operator described in Xilinx Floating-point Operator Version 5.0 [15].

**Table 1:** Xilinx IEEE-754 single-precision floating-point operator’s latency and slice register usage

**Floating-point operator**	**Low-latency**	**Slice registers**	**Max-latency**	**Slice registers**
fadd	3	139	12	547
fmul	3	87	8	361
fdiv	11	499	28	1377
fcomp	1	2	2	8

Since each PE has two independent 64-bit physical ports, we synthesized the PLF kernel to a deep-pipelined single-precision floating-point circuit with four input ports that receive four 32-bit data per cycle. The latency of the entire pipeline is 125 cycles.

### Hardware architecture of PLF kernel

**Figure 5:**
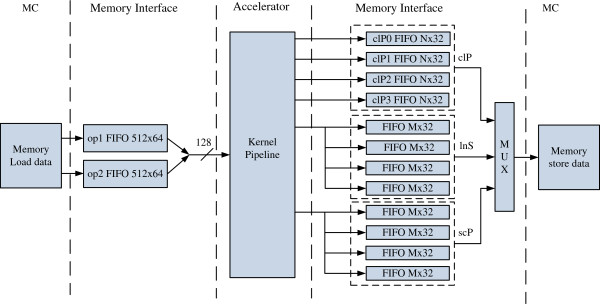
Hardware architecture of PLF accelerator.

The top level of the system design is shown in Figure 5. It is composed of a read/write memory interface and an accelerator. The kernel pipeline has four inputs and six outputs. Two 64-bit 512-entry input operand FIFOs *op1* and *op2* receive input data from the memory controller and feed four 32-bit floating-point input values per cycle to the pipeline. Six results from the pipeline are written into twelve FIFOs and from which the results are stored back to memory through a multiplexer. We chose 2048 and 512 for the output FIFO depth *N* and *M* respectively. The choice is determined by the constraint of the number of Xilinx BRAMs in a single FPGA [16] and BRAM usage of other modules and components.

In the write portion of the memory interface there are four FIFOs for clP, scP and lnScaler pipeline outputs. Four clP outputs (clP0, clP1, clP2, and clP3) correspond to four consecutive locations of clP array. By writing data into four FIFOs of scP and lnScaler in a round-robin manner, the memory interface can store 128 bits per cycle into the memory, increasing the output throughput.

**Figure 6:**
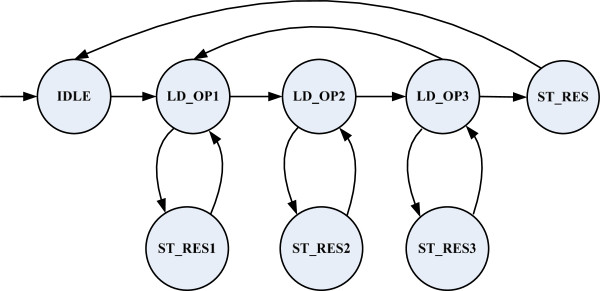
Memory access FSM.

Figure 6 shows a finite state machine (FSM) that coordinates memory read and write requests with Convey’s memory controller interface. In the *LD1* state, the controller requests two 64-bit words from the clL array, which comprises four 32-bit input values from the clL array. It then transitions to state *LD2* where it requests two 64-bit words from the clR array, which comprises the four 32-bit input values from the clL array. Next it transitions to the *LD3* where its requests a 32-bit word from the lnScaler array and the numSites array.

Until the output FIFOs have not reached full state, the controller repeats this sequence. When the output FIFOs become almost full, the current load request is interrupted and the machine state jumps to its store result state. The output results in the twelve FIFOs are then stored back to memory. When stores are finished, the interrupted load request will be resumed. Note that the pipeline can continue consuming input data if the input and output FIFOs are not full. After all load requests are satisfied the machine goes to the store state to store the remaining data in the FIFOs. When it is complete, the machine returns to the initial state *IDLE*.

### Steps of calculating root log likelihood

BEAGLE provides a set of interface functions needed for the user to describe a candidate tree and request that its likelihood be computed. Specifically, these include functions that:

•initialize input arrays and scalars, including the transition probability tables and leaf node (tip) conditional likelihood tables, and all other necessary scalars and arrays,

•describe the topology of the proposed tree,

•request that the BEAGLE runtime library traverse the tree and update the conditional likelihood tables of all internal nodes, and

•request that BEAGLE compute the likelihood of the tree.

**Figure 7:**
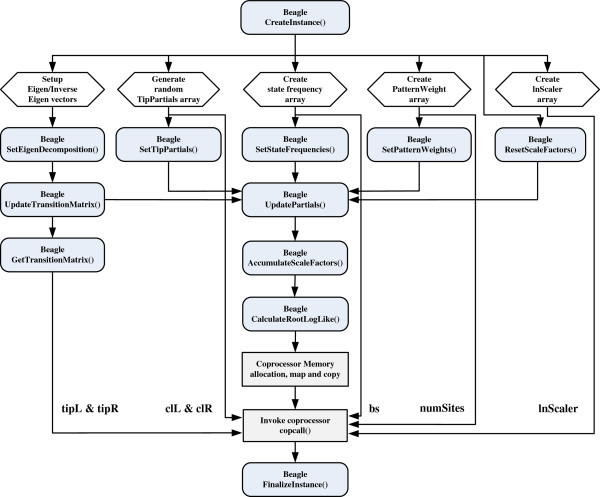
Mapping between BEAGLE API and coprocessor communication.

Figure 7 and Table 2 show how our driver program initializes all input data using a sequence of BEAGLE function calls.

**Table 2:** Descriptions of BEAGLE API implementation

**BEAGLE API call**	**Arrays initialized and coprocessor action**
*beagleSetEigenDecomposition beagleUpdateTransitionMatrix beagleGetTransitionMatrix*	4x4 transition probability matrices for each node (initialize arrays tipL and tipR in Pseudocode 1)
*beagleSetTipPartials*	Copy an array of partials into an instance buffer (initialize arrays clL and clR in Pseudocode 1)
*beagleSetStateFrequencies*	Copy a state frequency array into an instance buffer (initialize array bs in Pseudocode 1)
*beagleSetPatternWeights*	Set the vector of pattern weights for an instance (initialize array numSites in Pseudocode 1)
*beagleResetScaleFactor*	Reset a cumulative scale buffer
*beagleUpdatePartials*	Calculate partials for all internal nodes (compute array clP in Pseudocode 1)
*beagleCalculateRootLogLike*	Calculate log-likelihood of root node. (compute arrays lnScaler and scP in Pseudocode 1, calculate lnL in Pseudocode 1)

### Data organization

In order to utilize eight processing elements (PEs) on each FPGA we distribute the input data among four FPGAs evenly. We copy the host data arrays to the coprocessor’s memory space based on the organization and partition of coprocessor’s memory.

Convey’s memory mapping scheme requires that memory addressing of each PE be aligned to the MC controller number. Because of this, the data is arranged using a stride of 64 bytes for each PE in an AE and 512 bytes for the same PE space between two consecutive AEs. (e.g. AE0 and AE1).

**Figure 8:**
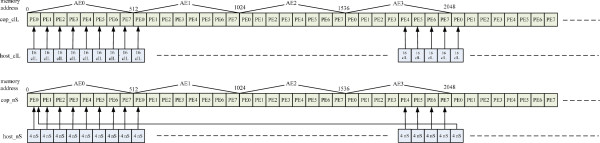
Memory allocation.

Figure 8 shows the memory mapping of the host array clL and numSites (nS). Each PE in an AE addresses 16 elements of clL array (64 bytes), which correspond to input data of four consecutive sites. Since there is only one input data of numSites for each site, we need to pack the next four numSites input data into PE0’s addressing space in AE0. This is shown in Figure 6 with an arrow from host array indexed at byte address 2048 to PE0 at byte address 0. The mapping of host array clR is the same as that of clL. We initialize the elements of lnScaler with zero.

## Results and discussion

We implemented the kernel on a multi-FPGA platform Convey HC-1. The platform has four Xilinx Virtex5 LX330 FPGAs. The design is described using our pipeline synthesis tool which generates deep floating-point pipeline in Verilog HDL. Xilinx ISE 13.2 [17] is used to synthesize, map, place and route the design, and generate the final bitstream. A single bitstream file is used to configure all the FPGAs. Each FPGA works on different input data based on the FPGA numbers and processing elements in each FPGA.

### Performance results of our implementations

In the software implementation using BEAGLE APIs, we timed the elapsed time of function *beagleUpdatePartials(), beagleAccumulateScaleFactors()* and *beagleCalculateRootLogLike()* since they comprise the computation kernel. In the hardware implementation, we timed the kernel execution on multi-FPGA. We assume the number of sites is a multiple of 128 to allow each PE to process the same amount of input data.

The performance results are shown in Table 3. When the number of sites is small, CPU implementation performs faster than both FPGA-based and GPU-based implementations. When the number of sites is larger than 512, both FPGA and GPU implementations outperform the CPU. For a large number of sites, we obtain around a 40X speedup compared to a single-threaded CPU implementation (Xeon E5520) and around 3X speedup compared to a many-core GPU implementation (Tesla S1070/T10). Note the GPU results are not available when the number of sites exceeds 260 K, since the BEAGLE GPU implementation is not capable of processing this data size.

**Table 3:** Performance results of our design

**nsites**	**CPU(us)**	**GPU(us)**	**FPGA(us)**	**Memory efficiency (%)**	**Speedup FPGA vs. CPU**	**Speedup FPGA vs. GPU**
128	27	93	96	2	0.28	0.97
256	41	93	101	4	0.41	0.92
512	69	94	103	7	0.67	0.91
1024	133	99	106	13	1.25	0.93
2048	225	107	107	20	2.10	1.00
4096	462	130	115	30	4.02	1.13
8192	944	167	125	28	7.55	1.34
16384	1894	240	148	28	12.80	1.62
32768	3873	385	207	28	18.71	1.86
65536	7922	672	304	29	26.06	2.21
131072	15898	1247	415	38	38.31	3.00
262144	31774	n/a	764	40	41.59	n/a
524288	63696	n/a	1240	44	51.37	n/a
1048576	127957	n/a	2280	46	56.12	n/a
8192000	1028649	n/a	15750	49	65.31	n/a

We achieved a memory efficiency of around 50% for large data sizes. This is competitive with similar implementations in the literature. For example, Cong et al. reported their implementation of a bandwidth-bounded application that has 32 PEs and utilizes all the memory access ports on Convey HC-1 [18] having 30% efficiency. In general, the factors that contribute to the efficiency are external memory access order, memory buffer size, and frequency of memory bus turns (alternating between read and write operations).

Convey’s unique interleaved memory addressing attempts to maximize memory system utilization by distributing memory accesses across all memory banks. Memory stalls occur when the number of pending memory load requests reaches the size of memory request queue in the memory controller. In order to avoid this, the size of the custom memory buffer in the user design must be close to the size of the request queue. A smaller memory buffer cannot overcome the long latency of DDR2 memory access while a larger one increases memory stalls.

Frequently alternating between memory read and memory write will reduce the effectiveness of the Convey memory scheduler and reduce memory bandwidth. The use of deep output FIFOs and writing the entire contents of the output FIFOs when they fill will reduce the frequency of read-write transitions and improve bandwidth.

### FPGA resource usage

The resource utilization of each FPGA is listed in Table 4. In each PE all floating-point multipliers are implemented using DSP48E modules [19] while other floating-point operators are implemented using LUTs. Due to the large number of floating-point operators and deep pipeline circuit in each PE we utilize nearly all the slices in a single FPGA. The design runs at 150 MHz and the memory controller at 300 MHz.

**Table 4:** Area results of our design

**Resource**	**Utilized**	**Total available**	**Utilization ratio**
LUT	193,518	207,360	93%
FF	200,833	207,360	97%
Slice	51,629	51,840	99%
BRAM	235	288	82%
DSP48E	152	192	79%

### Power consumption

The Xilinx power analyzer *xpa* reports that each FPGA design consumes about 23 W, or 92 W for all four FPGAs. The thermal design power of the Tesla GPU card is around 200 W. The FPGA implementation delivers a better performance while consuming less than half of the power of the Tesla GPU.

## Discussion

### Design motivation

This work is based on the “MrBayes accelerator” design, previously developed in the author’s lab [8]. The original design performed the same basic computations as described in this paper, but its pipeline was designed by hand and did not incorporate any functional unit reuse. As such, the original design instanced one functional unit for each operator in the DFG. The resulting design was large, allowing only one PE to fit on a single FPGA. In order to automate the design process and improve the resource efficiency, the authors developed a high-level synthesis tool that generates a pipeline from a data-flow graph description of the kernel, and exploits functional unit reuse in such a way as to achieve the maximum throughput as bounded by the available memory bandwidth on the target platform. This synthesis tool was developed specifically for this application, but can be also used for any data-intensive kernel that has no loop-carried dependencies.

In addition, we implemented the new version of the design on the Convey HC-1 reconfigurable computer, which has 13.2 times the amount of FPGA resources, 28.4 times the memory bandwidth, and over 1000 times the memory capacity of the original platform, an Annapolis Micro Systems WildStar II Pro. In order to make the design more general purpose, we integrated our design with the BEAGLE library instead of integrating it only into MrBayes 3 as in the original work.

### Performance results

Our design achieves the highest possible level of performance as allowed by the memory system of the HC-1. Memory efficiency was relatively low, and can be potentially improved by rearranging the order in which inputs are requested from the memory. Specifically, this can be performed by buffering a set of consecutive values from each input array before streaming the values into the pipeline in the order implied by the outermost loop in Pseudocode 1. This would require that the input values be read from memory in a different order than read by the pipeline. While we expect this to improve memory efficiency, the buffer would need to be designed in a way that doesn’t itself contain inefficiencies that negate its benefit. This is part of the authors’ future work.

## Conclusions

In this paper we described an FPGA-based implementation of the core computations in the BEAGLE library that perform the phylogenetic likelihood function and tree likelihood computations. With this design we achieve 3X the performance of BEAGLE’s GPU-based implementation for large datasets.

The kernel implemented in this work is characterized by having a relatively low arithmetic intensity, making its performance dependent on the effective memory bandwidth achievable by the target platform. In order to achieve high performance under this condition, we developed a design automation tool that synthesizes the kernel’s data flow graph in a way that matches the pipeline’s throughput to the platform’s memory bandwidth while minimizing hardware requirements.

## Availability and requirements

**Project name:** BEAGLE_HC1

**Project home page:**http://www.cse.sc.edu/~jbakos/software.shtml

**Operating system(s):** Linux

**Programming language:** C and Verilog

**Other requirements:** Must be run on a Convey HC-1

**License:** GNU GPL v4

**Any restrictions to use by non-academics:** None

## Competing interests

Both authors declared that they have no competing interests.

## Authors’ contributions

JZ implemented the high level synthesis tool used to synthesize the BEAGLE kernel onto the Convey HC-1 platform. JZ verified the design and performed performance testing. This work was performed under the direction of JB (JZ’s research advisor), whose original design, implemented on another FPGA platform [8], was the motivation and starting point for this improved design and design methodology. The manuscript was written jointly by JZ and JB. Both authors read and approved the final manuscript.
